# A Multi-Channel Opto-Electronic Sensor to Accurately Monitor Heart Rate against Motion Artefact during Exercise

**DOI:** 10.3390/s151025681

**Published:** 2015-10-12

**Authors:** Abdullah Alzahrani, Sijung Hu, Vicente Azorin-Peris, Laura Barrett, Dale Esliger, Matthew Hayes, Shafique Akbare, Jérôme Achart, Sylvain Kuoch

**Affiliations:** 1 Wolfson School of Mechanical, Manufacturing and Electrical Engineering, Loughborough University, Ashby Road, Loughborough, Leicestershire LE11 3TU, UK; E-Mails: A.Alzahrani@lboro.ac.uk (A.A.); V.Azorin-Peris@lboro.ac.uk (V.A.-P.); 2 National Centre for Sport and Exercise Medicine, School of Sport, Exercise and Health Sciences, Loughborough University, Ashby Road, Loughborough, Leicestershire LE11 3TU, UK; E-Mails: L.A.Barrett@lboro.ac.uk (L.B.); D.Esliger@lboro.ac.uk (D.E.); 3 Cambridge Consultants Limited, Science Park, Milton Road, Cambridge CB4 0DW, UK; E-Mail: matthew.hayes@cambridgeconsultants.com; 4 Université Paris-Sud 11, Polytech’Paris-Sud–Service des stages, Bât 620, Orsaycedex 91405, France; E-Mails: shafique.akbare@gmail.com (S.A.); achart.j@gmail.com (J.A.); sylvain.kuoch@gmail.com (S.K.)

**Keywords:** multi-channel opto-electronics, accelerometer, motion artefact, sport physiological monitoring, heart rate, pulse wave, vital signs, wearables

## Abstract

This study presents the use of a multi-channel opto-electronic sensor (OEPS) to effectively monitor critical physiological parameters whilst preventing motion artefact as increasingly demanded by personal healthcare. The aim of this work was to study how to capture the heart rate (HR) efficiently through a well-constructed OEPS and a 3-axis accelerometer with wireless communication. A protocol was designed to incorporate sitting, standing, walking, running and cycling. The datasets collected from these activities were processed to elaborate sport physiological effects. t-test, Bland-Altman Agreement (BAA), and correlation to evaluate the performance of the OEPS were used against Polar and Mio-Alpha HR monitors. No differences in the HR were found between OEPS, and either Polar or Mio-Alpha (both *p* > 0.05); a strong correlation was found between Polar and OEPS (r: 0.96, *p* < 0.001); the bias of BAA 0.85 bpm, the standard deviation (SD) 9.20 bpm, and the limits of agreement (LOA) from −17.18 bpm to +18.88 bpm. For the Mio-Alpha and OEPS, a strong correlation was found (r: 0.96, *p* < 0.001); the bias of BAA 1.63 bpm, SD 8.62 bpm, LOA from −15.27 bpm to +18.58 bpm. These results demonstrate the OEPS to be capable of carrying out real time and remote monitoring of heart rate.

## 1. Introduction

People suffering from cardiovascular disease (CVD) need to observe their health conditions continuously in order to prevent any further deterioration, to determine current health status [1] and to maintain patients’ quality of life [2]. In today’s growing and ageing population, cardiovascular disease, stroke and diabetes are the main causes of disability and death [3]. Home and Tele-monitoring can be a cost-effective solution in forthcoming healthcare, and could help elder and illness people maintaining their quality life [4]. Tele-monitoring together with home-delivered care is an inevitable trend and becoming a valuable means to reduce the costs of treatment and to increase service quality in our routine healthcare world [5].

**Figure 1:**
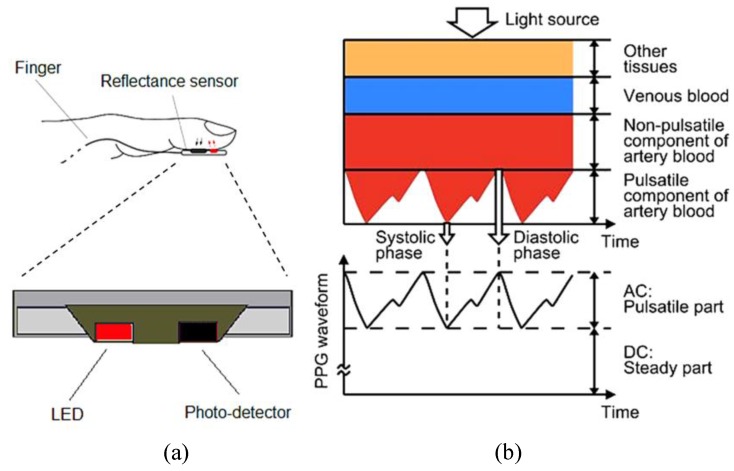
Illustrated diagram of the reflectance sensor (**a**) and the light absorbance of tissue components (**b**) (revisited and modified from [19]).

Monitoring of physiological parameters such as heart rate (HR) is also crucially important in many sports and exercise applications in order to monitor training and to ensure athletes are training at the right intensity. In addition, monitoring HR and heart rate variability (HRV) could potentially play a vital role in the prevention and detection of overtraining [6]. HR monitoring is also used as a means of objectively monitoring physical activity levels and as a means of estimating energy expenditure [7]. Photoplethysmography (PPG) is a non-invasive optical technique used to measure blood volume change in micro-vascular beds of tissue [8]. Photoplethysmography can be used to monitor health vital signs, such as HR, HRV, respiration rate (RR), blood pressure (BP) and oxygen saturation (SpO_2_%) [9]. The principle of detection can be described with Lambert Beer’s law to predict the intensity of red and IR light transmitted through and reflected/refracted from a pulsatile area of the body, such as a finger, forehead, in-ear or wrist onto a photo detector [8,10]. Figure 1 shows the absorption of pulsatile tissues as AC and non-pulsatile as steady DC absorption.

Wearable health monitors has recently become a popular and interesting topic among researchers and commercial companies. Many wearable devices such as FlexPock device [2], MAIN Shirt (MAIN: short for magnetic induction) [4], AMON (Advanced Medical Monitor) physiological monitor [11], Nonin Wrist Ox3100 (Nonin Medical, Inc., Plymouth, MN, USA) [12] SenseWear (Pittsburgh, PA, USA) [13], Smart Shirt (Sensatex Inc., Bethesda, MD, USA) [14], LifeShirt (Vivometrics Inc., Ventura, CA, USA) [15], and Smart Vest (Electromed, Simi Valley, CA, USA) [16] have been developed with various structures and electronic systems. Many of the devices are yet to provide a high performance incorporated with, and real time monitoring functionalities during an extensive movement >8 km/h. For example, AMON wrist-wear is utilized for high risk patients [17] in bed or during normal walking, such as with elderly people, but not for jogging. Moreover, the data generated from Nonin Wrist Ox3100 [12] band is processed offline, as the data are stored locally in non-volatile memory and are extracted and analyzed at a later stage. This could lead to delays in diagnosis and is considered a limitation. Despite their wealth of physiological information, reliable PPG signals can be subject to heavy interference originating from different sources, including modification of the optical properties of the internal tissues, poor blood perfusion, and external sources such as electromagnetic and electronic noise, physical movement, misalignment between skin surface and optical sensor path and ambient light. Some artefact noises could be dealt with pre-processing though utilization of digital filters and ambient light subtraction approach as one of major challenges in PPG signal processing is motion induced error. In this case, motion artefact can influence the measurement signal and lead to a disrupted readout [8]. Lee *et al.* [18] indicated that the greatest and worst cause of artefact noise that contaminates the PPG signals is motion artefact (MA) produced from physical activity and body movement. Thus, various signal processing techniques have been proposed to remove MA and recover the PPG signals.

When noise is out of the range of physiological signals, average filtrating [20] is a filtration technique usually used to remove the noise. However this technique sometime cannot work effectively when the noise is near or shares the same frequency band as the signal components of interest as this technique can suppress the desired signals. Another approach is independent component analysis (ICA) as recommended by Yoo *et al.* [20] to use a block interleaving and basic ICA algorithm. Natarajan *et al.* [21] proposed an application of ICA in the frequency-domain as it is assumed that the subcomponents are non-Gaussian signals and are statistically independent from each other, thus the ICA does not capture PPG signals contaminated by MA. The periodogram method [22] has been used to estimate the HR with some drawbacks of inconsistent spectrum, high variances as well as serious leakage effects [23].

The adaptive noise cancelation (ANC) was also proposed to remove the motion artefact as Ram *et al.* [24] and Yousefi *et al.* [25] suggested using ANC to eliminate MA. To implement ANC, the system needs a higher computational memory, and the performance of ANC is sensitive to a reference signal. Among these techniques, the acceleration data (AD) could be considered at present as one of operative techniques to reduce MA. Fukushima *et al.* [26] suggested using a spectrum subtraction method to cancel the spectrum of acceleration data from the desired PPG signal. The AD with Kalman filtering (KF) as indicated by Lee *et al.* [27] could also reconstruct the signal from MA. Further techniques to eliminate or reduce MA include empirical mode decomposition (EMD) [28], electronic processing methodology [29], evaluation wavelet [30], and time-frequency analysis [31]. Despite the importance of signal processing, these methods cannot be used in many scenarios due to their implicit assumption that the corruption manifests itself as an additional signal component unrelated to physiological status either in time, frequency or statistical domains [32]. Most of these techniques were proposed and carefully set up for specific scenarios when users performed designated instructions with specific motions, e.g., keyboard typing, finger movements [23,24,33] and walking [25]. Thus these techniques might perform well when users make intensive physical movements such as those made during exercise. In addition, some studies were conducted by Poh *et al.* [34] and Lopez *et al.* [35] aimed to reduce MA and estimate HR in the presence of movement. Both studies focused on slow running at speed <8 km/h while Lopez *et al.* [35] utilized the earlobe as the site to detect PPG signals, where less MA is exhibited. Hence, the above studies have merely concentrated on physical activities under constraints of slow movement.

A multi-channel opto-electronic patch sensor (OEPS) [36] was employed in the study. OEPS is the outcome of opto-physiological interaction work to specifically study the effluence of tissue optical properties on blood driven dynamic changes named as opto-physiological modelling (OPM) [37]. An optimal optical sensing layout together a plurality of light sources with one photodetector configured to capture radiation after it has passed through the tissue is one of key factors to effectively capture these dynamic changes and the OEPS [38] was led by the OPM driven photoplethysmographic measurement studies [39,40]. Hence the OPES comprises of the following aspects:

1. The tissue optics properties to determine an optimal optical sensing position;
2. An optimal optical layout for the OEPS operable to monitor the tissue optic properties of the tissue type; and
3. Optical design involving in the options of a wavelength, intensity and an optical path length for the LED illumination sources.

Meanwhile, an analogue three-axis MEMS accelerometer (3MA, ADXL337, Analog Devices Inc., Norwood, MA, USA) was employed to detect acceleration and movement in all three axes with respect to gravitational acceleration and to provide a reliable movement reference. The 3MA provides higher accuracy in the characterization of physical movement with its three signal outputs for accelerations along spatial vectors X, Y and Z. The value of 3MA stems from the fact that capacitance changes inside the accelerometer reflect forces on all three axes in respect of gravitational acceleration. The total acceleration vector is produced as a combination of vectors X, Y and Z. When the user’s body moves, the accelerometer produces signals proportional to the magnitude of the movement, and the resultant total acceleration vector R represents the instantaneous direction and magnitude of motion in 3D space.

The purpose of this study was to assess HR by means of OEPS with sixteen healthy subjects across a range of physical activities, e.g., resting, cycling, jogging and running. A customized OEPS was attached to different measuring sites of the subject, e.g., palm, finger, forehead and back where the wearers perform intensive physical activities such as running up to 14 km/h as well as cycling against various weight loads up to 2.5 kg*.*

## 2. Method

### 2.1. Experimental Setup of Opto-Electronic Patch Sensor (OEPS)

**Figure 2:**
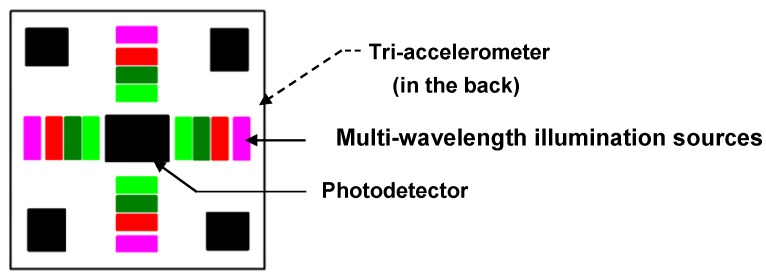
Schematic diagram of Opto-Electronic Patch Sensor (OEPS) with accelerometer.

The OEPS was constructed as a reflection opto-electronic sensor along with the outcome of opto-physiological modeling together with a specific optoelectronic system to reduce the impact of motion disturbance. The optimal optical design for the OPES is shown in Figure 2.

The optimum separation of each of a plurality of light sources from a photodetector, based on modelled optical path lengths for light travelling from each light source, through the body tissue type to be monitored, to the photodetector; and locating light sources of different wavelengths at different distances from the photodetector. A reasonable distance between illumination sources and photo-sensor is required in order to avoid a saturation signal from a closer source and a reduced amplitude signal from a far light source [2]. A non-invasive OEPS is ideally configured for use with a body tissue type to be monitored so as to allow optical data, relating to the physiological parameters being studied, to be reliably and accurately obtained.

**Figure 3:**
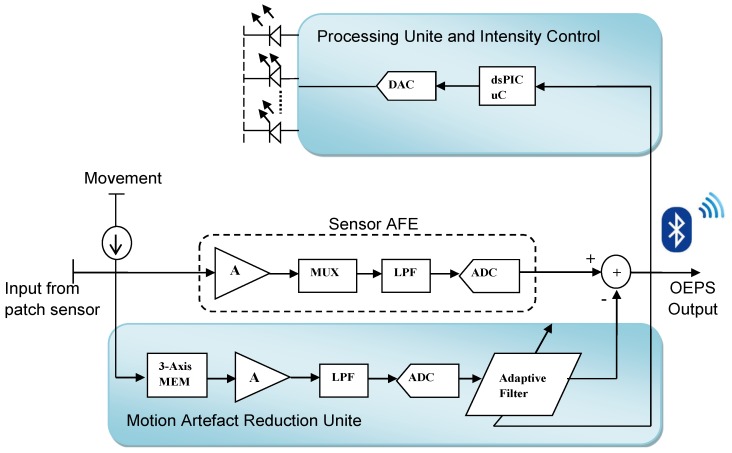
Electronic system structure of OEPS for continuous physiological monitoring.

High gain, low noise and ultra-low power consumption in the OEPS electronic system was determined for continuous and real time monitoring. In Figure 3, the OEPS system shows a wearable health monitoring system wirelessly connected via a Bluetooth module to a Bluetooth-enabled cell phone/PC to perform further signal processing and display the outcomes of measurement.

Figure 3 illustrates the flow of OEPS electronics system. The output from the OEPS is passed through the analog front end (AFE). In the end, normal PPG signals are usually sent through a Bluetooth module. In the presence of movement, the module of motion artefact reduction (MAR) takes an execution action against a motion as the system structure takes the corrupted signals gained from the OEPS as a primary input and the motion signals from the 3-axis Micro-Electro-Mechanical Systems accelerometer (3MA) as a reference input.

To reach such an accurate level, the vector R of the accelerometer motions is mapped to the contribution of motion artefact. Actually, the accelerometer is most sensitive when the sensing axis is close to 0*°* and less sensitive when the sensing axis is near 90*°*.

**Figure 4:**
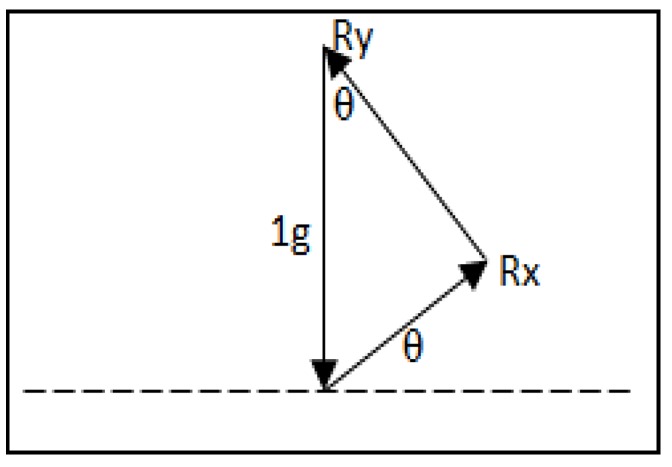
The combination of *x* and *y* vectors in two axis acceleration.

Hence, the sensitivity could be preserved and compensated when combining *x* and *y* vectors as shown in Figure 4.

The combination of the vectors is equal to the output values of acceleration which are the projection of the force gravity as expressed on Equation (1)*.*

(1) $$R=\;\sqrt{Rx^{2}+Ry^{2}}$$

**Figure 5:**
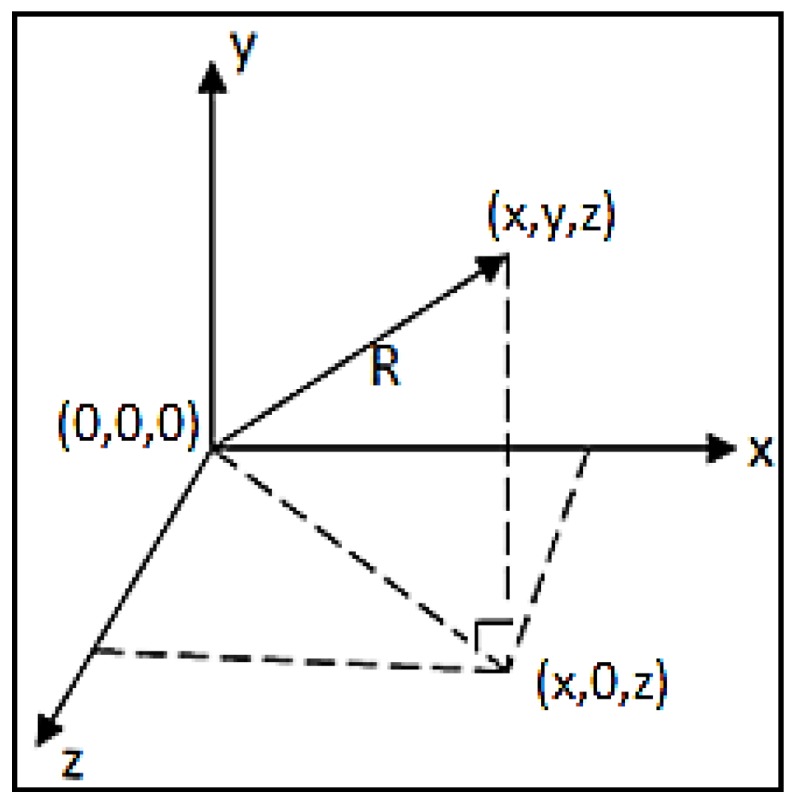
The combination of *x,y* and *z* vectors in three axis acceleration.

In this study, 3MA accelerometer was used to generate an accurate calibration of acceleration since the three vectors *x*, *y* and *z* were presented. The total acceleration vector is shown in Equation (2) and the combination vectors of x, y and z is shown in Figure 5.

(2) $$R=\;\sqrt{Rx^{2}+Ry^{2}+Rz^{2}}$$

Thus, an effective cancellation method was introduced to reduce motion artefacts on corrupted PPG signals using the reference signals from the 3MA, followed by an adaptive filter to facilitate the optimum adaptive motion artefact cancellation technique as seen in Figure 3. To realize the adaptive noise cancellation, the OEPS system uses two inputs, e.g., the corrupted PPG signal as a primary input, and the combined motion signal of the accelerometer as a reference input. The motion signal is deducted from the primary input by the means of a close loop least mean square LMS adaptive filter to adjust its variable coefficients until the error is minimized to yield a recovered output signal since there is a difference between the output from the filter and the signal as desired. The adaptive filter for reducing motion artefacts was set to a filter order of 32 and a step size of μ:0.1.

**Figure 6:**
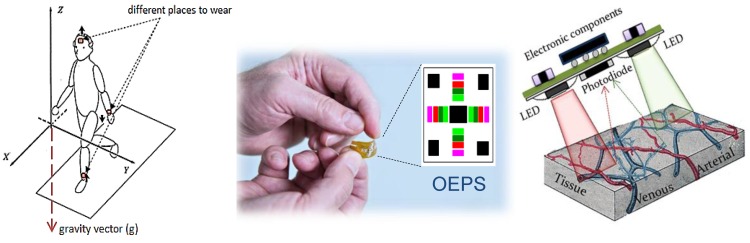
Flexible OEPS for continuous physiological monitoring and its viable locations.

The OEPS consists of (1) an optical side to catch the backlight containing a serial of pulsatile signals; and (2) the electronic side with an analogue front-end to drive multi-wavelength LEDs and to pre-amplify the photodiode from (1), and the embedded digital for signal processing procedure and a communication module to wirelessly transmit the physiological outcomes to a computer and smartphone. The OEPS operates in reflectance mode (*i.e*., multi-wavelength illumination sources and the detector are on the same side) to make it suitable for attachment or adherence onto different locations of a human body, *i.e.*, forehead, palm, earlobe and wrist, as presented in Figure 6. However, the amplitude of pulsatile signal in a reflectance mode is smaller than a conventional transmission mode; thus, a trans-impedance is required in order to convert photocurrent intensity into a proportional suitable voltage to enhance the signals generated from the photodiode [5].

Multi-wavelength illumination sources, *i.e.*, LEDs of green 525 nm, red 650 nm and IR 870 nm (JMSienna Co., Ltd., Palo Alto, CA, USA) were used for the OEPS including, a low-profile PiN photodiode (BPW34SR18R, Osram, GmbH). The analogue front-end consists of a pre-amplifier, a multiplexer and a low pass filter to performer-processing of the analogue signals gained from the OEPS to be prepared for analogue-to-digital conversion (ADC). A3MA (ADXL337, Analog Devices Co., Palo Alto, MA, USA) was employed to detect any motion from the body and the signal generated from the 3MA was used as a reference against MA. A dsPIC Microcontroller (dsPIC33FJ64GS610, Microchip Co., Chandler, AZ, USA) was hired to implement digital signal processing (DSP) and communication via a Universal Asynchronous Receiver/Transmitter (UART) protocol.

### 2.2. Multiple Wavelength Illumination Source

**Figure 7:**
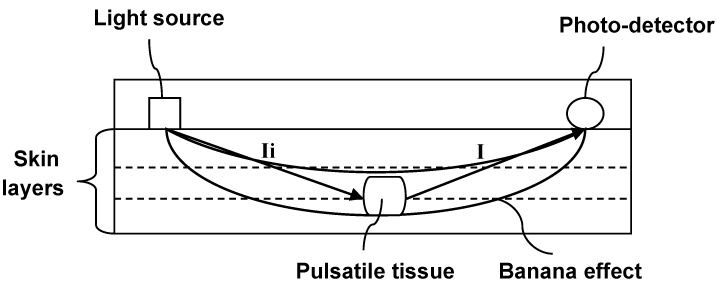
Banana shape effect on reflection mode OEPS.

Since there is a variation in depth of penetration and absorption over the range of wavelengths [41], the intensity of the light penetrating through the skin and emerging from the tissue can be represented as the banana shape effect. Figure 7 shows the light path distribution of typical PPG model. *I_i_* is the light incident on the skin surface; *I*_0_ is the intensity of light penetrating into the skin, *I_r_* is the reflected light intensity and *I* is the intensity of light emerging from the tissue through the banana effect. Referring to the principle of Lambert-Beer’s law, the light intensity passing through different layers of skin is decayed exponentially.

The light back to the photo-detector can be separated into a non-dynamic (DC) component that results from non-pulsatile tissues and a dynamic (AC) component due to the presence of pulsatile blood volume changes. Thus, the model can be expressed as:

(3) $$I=Ii\times e^{-\text{λ}t}=Ii\times e^{-\left({\text{μ}}_{eff}\;\times \;r\right)t}$$

where λ is the wavelength of a particular light, *I* is the reflected light intensity after penetration, *Ii* is light incident, μ*_eff_* is the absorption coefficient of both dynamic and static components, and r is the light path length. Equation (3) can also be expressed as following formula

(4) $$I=Ii\times e^{-\left({\text{μ}}_{dy}\;\times \;d\left(t\right)\;+{\text{ μ}}_{st}\;\times \;m\right)}$$

where μ*_dy_* is the absorption coefficient of the dynamic and μ*_st_* is the static coefficient, the light path length through the dynamic and static components are *d(t)* and *m*, respectively. The target is to enhance the pulsatile dynamic variation (PDV) upon different wavelengths and through multiple layers of the skin. The accuracy of PDV depends upon the quality of the AC component; however, the gain of PDV over the tissues (finger, ear-lobe) never exceeds 5% of the incident light intensity [40]; even using super bright LEDs, the PDV light intensity remains on low values or could saturate the photo-detector (PD) due to the increase of reflected light *I_r_*. Therefore, the *PDV* should be maximized and this could be achieved by maximizing the light that travels or penetrates through the tissue *I* and minimizing the reflection component of the static signal *I_r_*. The light intensities at the PD can be expressed as:

(5) $$It=\left(Ir+Io\right)\times e^{-\left({\text{μ}}_{dy}\times d\left(t\right)+{\text{μ}}_{st}\;\times \;m\right)}$$

Expanding Equation 5 and splitting the dynamic and static term results in Equation (6).

(6) $$It=\left(Ir+Io\right)\times e^{-{\text{μ}}_{st}\;\times \;m\;\left(1\;+\;{\text{μ}}_{dy}\;\times \;d\left(t\right)\right)}$$

By applying a band pass filter with the cut-off frequency 0.18 Hz *< f <* 10 Hz as in the design of *OEPS,* as a result any constant value and the *I_r_* can be eliminated. By normalizing the dynamic component with respect to the static component, Equation (7) can be used as the dynamic component becomes independent of the light source intensity.

(7) $$\frac{AC}{DC}=\frac{Io\times {\text{e}}^{-\left({\text{μ}}_{st}\;\times \;m\right)\;\times \;{\text{μ}}_{dy\;}\times \;d\left(t\right)}}{Io\times {\text{e}}^{-\left({\text{μ}}_{st}\times m\right)}}={\text{ μ}}_{dy}\times d\left(t\right)$$

To obtain the optimum value of PDV*,* the absorption light is tested at the lab with multiple wavelengths and various distances between the peak of incidence light and the active area of the -detector. According to Lee [42] the green light PPG was found to have relative robustness against motion artefacts as compared with red and blue light PPG. Hence, the green wavelength is presented in this study as being more robust than the red and IR wavelength with the presence of movement.

**Figure 8:**
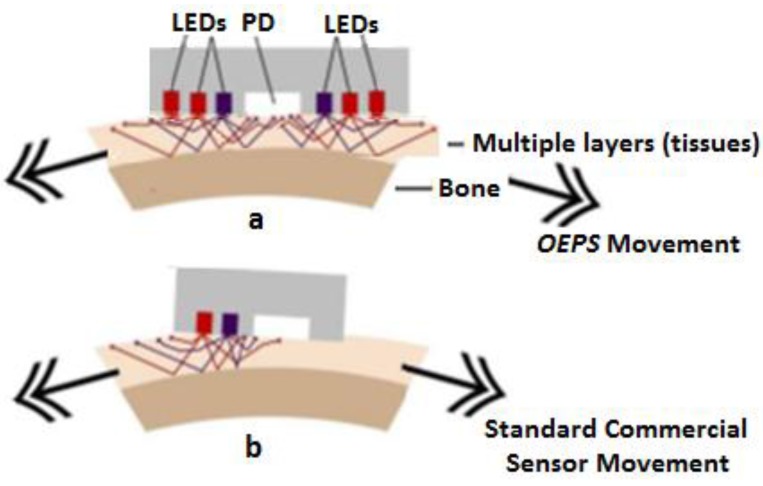
Sensor displacement altering backscattered light.

A multiple controlled LED modulator was created to stabilize the output of illumination of the *OEPS* patch sensor and to enhance the back-scattered light signals that are detected as seen in Figure 6. When the user moves in a random way the PVD can still be captured. Moreover, the OEPS can handle displacement and misalignment as indicated in Figure 8a. OEPS typically captures back-scattered light with movement because the OEPS can capture from other mirrored wavelength elements when one of the elements is being misaligned with the vessels. In contrast, Figure 8b shows a standard commercial probe movement inducing and causing changes in the sensor position, changing the detected backscattered light. Additionally, the 3MA present in the system was used as a reference to reduce motion artefact [38].

### 2.3. Physiological Monitoring Protocol

Sixteen healthy active male participants (aged 24 ± 3 years, mean ± standard deviation) volunteered to take part in the present investigation. The protocol was approved by the Loughborough University Ethical Advisory Committee and implemented in the sport physiological measurement laboratory in School of Sport, Exercise and Health Sciences, Loughborough University. Prior to any tests all participants had the procedures and possible risks associated with involvement in the study explained to them and their written consent to participant in the study was obtained. The measurements were taken while the participants were seated, while they walked and ran on a treadmill and cycled on a cycle ergometer. Gender (Male), Height (179.8 ± 4.1 cm), body mass (74.9 ± 7.9 kg),body mass index (23.1 ± 2.0 kg/m^2^) and blood pressure (systolic 130 ± 10 mmHg, diastolic 67 ± 9 mmHg) for individual participants as well as the room temperature (24.4 ± 0.5 °C) and humidity (33% ± 2%), were also measured during the experimental procedure.

HR was simultaneously monitored with a Polar Bluetooth® Smart chest strap ECG device, an accessory to Polar’s Bluetooth® Smart wGT3X-BT activity monitor (Acti-Graph, LLC, Pensacola, FL, USA) as well as Mio-Alpha (Physical Enterprises Inc., Vancouver, BC, Canada) wrist watch HR monitor as the references. The back of OEPS sensor attached with 3MA was placed on the palm of the participant’s left hand while the Mio-Alpha was worn on the left hand wrist. Both the OEPS and the commercial devices (Mio-Alpha and Polar) captured data simultaneously to facilitate the processing signals of and reference indications respectively. The participants were asked to rest in a seated and upright position for 180 s, and the HR was then recorded over a 60 s period. Once the session of resting was completed to obtain HR, the participant started the exercise protocol on the treadmill (Technogym Excite Med 700, Gambettola, Italy).

**Figure 9:**
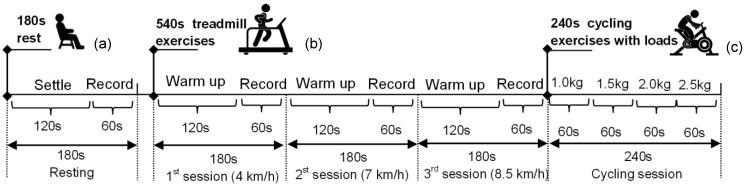
The illustrated protocol implementation of (**a**) resting on the chair with a period of 180 s; (**b**) walking and running on treadmill with an execution period of 540 s; and (**c**) cycling exercise with loads implemented in period of 240 s.

The protocol consisted of (a) resting on the chair with a period of 180 s; (b) walking and running on treadmill with an execution period of 540 s; and (c) cycling exercise with loads implemented in period of 240 s. The protocol for walking and running on treadmill composed of 1st session of walking exercise at a speed of 4 km/h within the period of 180 s; 2nd session of running exercise at a speed of 7 km/h within the period of 180 s; and 3rd session of faster running exercise at a speed of 8.5 km/h within the period of 180 s. The HR was detected by the OPES while the participant moved his left arm freely in each sessions including the resting and the walking and running. The participant was then asked to complete the cycling protocol consisting of four sessions (60 s each) cycling at the same speed of at 60 rpm on a Gym cycle (Monark Ergomedic 874E, Vansbro, Sweden) with four mass loads, for instance. 1st session with 1.0 kg, 2nd session with 1.5 kg, 3rd session with 2.0 kg, and 4th session with 2.5 kg. The HR was recorded in each session. Figure 9 represents the entire exercises protocol.

### 2.4. OEPS Measurement System

**Figure 10:**
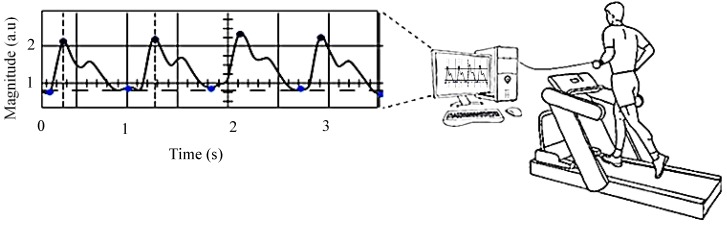
Physiological testing setup and platform, left is the LabView GUI and right is the treadmill.

The PPG signals were captured during the experimental measurements, and the system computed the HR every 15 second interval. All data sets from the OEPS were collected by the means of a 4-channel PPG board (DISCO4, Dialog Devices Ltd., Reading, Berkshire, UK). The ADC for these captured PPG signals was implemented by a 14bit data acquisition board (DAQ, USB-6009, National Instruments Co., Novato, CA, USA), and the control software of PPG board was performed by LabVIEW GUI (National Instruments Co., USA). The sampling frequency of 256 Hz was higher enough to reconstruct PPG signals within the Nyquist frequency condition, as well as the band pass filter of cut-off frequency 0.1–10 Hz were implemented to narrow down the region of interest of the signal. The desired signals were measured and PPG real time signals displayed on the screen of a PC as a waveform chart as shown in Figure 10.

## 3. Results

### 3.1. Data Analysis of HR Detection

**Figure 11:**
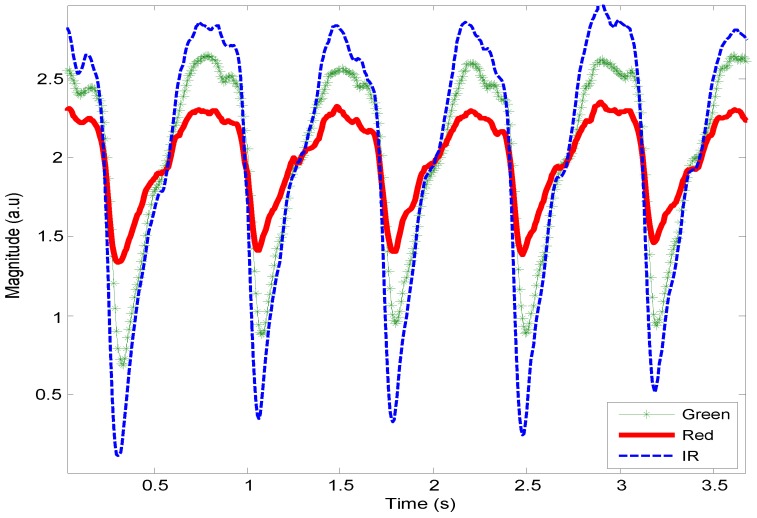
Photoplethysmography (PPG) signals captured by the OEPS with three wavelength illuminations*.*

Primary signal processing of the recorded datasets were executed using the Matlab software (The MathWorks, Inc., Natick, MA, USA) to evaluate the performance of the OEPS and commercial devices, for example, the Polar chest strap and Mio-Alpha wrist HR monitors. Two channel PPG signals from the OEPS and the acceleration reference were recorded by the execution program of LabVIEW. Figure 11 shows PPG signals captured from the palm attaching the OEPS with three wavelength illumination sources (*i.e*., green 525 nm, red 650 nm and IR 870 nm) during the stationary state where the participant was in the sitting position.

**Figure 12:**
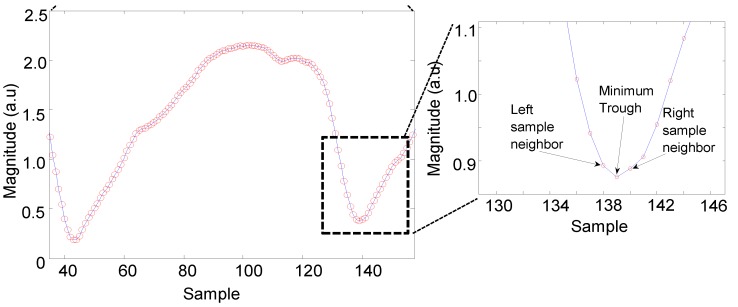
The algorithm of PPG peak and trough-detection (APTRD) developed to detect the trough of pulsatile waveform for HR calculation.

HR can be extracted by an algorithm by separating the raw signals of three different wavelength illuminations and even perform pulse-pulse interval PPI from an individual wavelength illumination. The algorithm of PPG peak and trough-detection (APTRD) was developed with MatLab (The MathWorks, Inc., Natick, MA, USA) to extract pulsatile waveforms as PPG AC components resulting in the calculation of HR. Figure 12 shows the APTRD how to work out the trough detection from a single wavelength illumination. The algorithm runs to analyze each sample along with the duration period and to decide which samples are smallest than the nearest two neighbor samples. HR can be determined through the minimum point detected between two minimum troughs.

**Figure 13:**
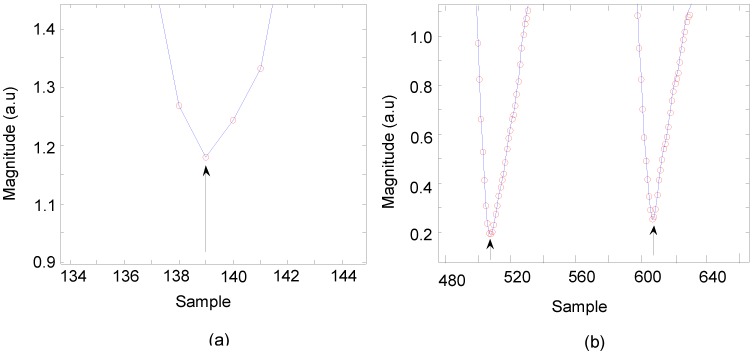
APTRD developed to detect a minimum valley of pulsatile features (**a**) at 139; (**b**) at 508 and at 607.

The APTRD runs on the sample period and detects the minimum trough samples by setting three amplitude values and comparing them, previous value, current value and next value. Figure 13 shows a close look of different random sample intervals in waveforms. The results obtained from the preliminary processing procedure of trough detection values are well matched with the waveforms as presented in Figure 13a,b.

**Figure 14:**
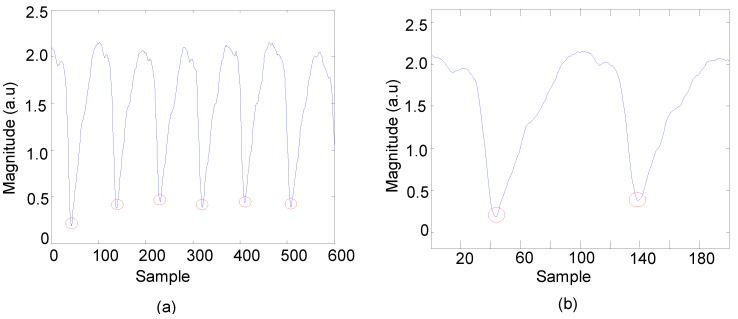
Inter beats count and average heart rate (HR) measurements from the pulsatile features of PPG signal. (**a**) Duration of 600 samples was detected as 6 beats count with 77bpm; (**b**) Duration of 180 samples was counted 2 beats with 77bpm.

The resulting inter beats count derived from the APTRD and the average HR measurements were calculated over different epochs for the same pulsatile features of PPG signal as seen in Figure 14a,b.

Figure 14a indicates the beat account in duration samples of 600 samples is 6 beats and the HR is calculated as 76.8000 ≈ 77 beats per minute (bpm). The algorithm was applied in different durations as displayed in Figure 14b and found that the beat account is 2 beats and the HR is 76.8000 ≈ 77 bpm.

**Figure 15:**
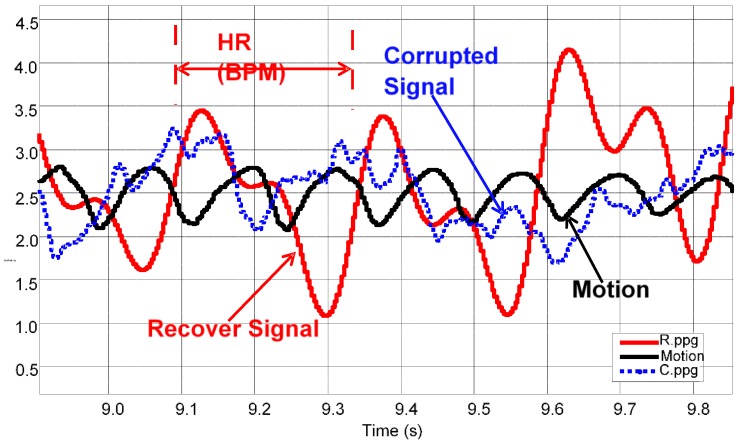
Recovery of PPG signals using motion artefact reduction (MAR).

In the presence of movement, the PPG signal starts to get corrupted and as a result of this it becomes difficult to obtain physiological information. Motion artefact reduction (MAR) [32] was primarily introduced to recover the PPG signal and reduce motion artefact. Figure 15 shows the corrupted signal (Blue), recovered signal (Red) and reference 3MA signal (Black).

**Figure 16:**
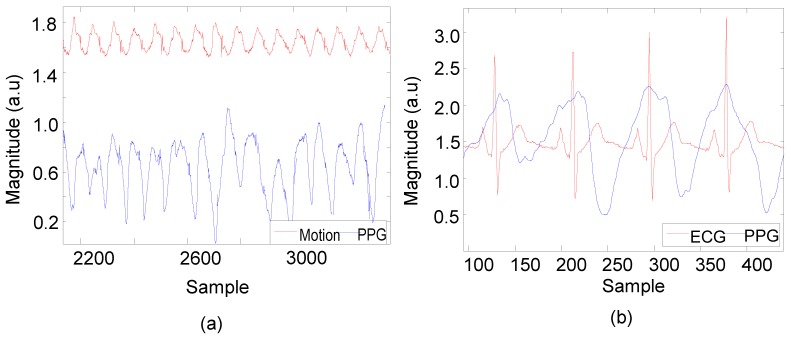
Recovery of PPG signals through motion artefact reduction (MAR) and synchronizing these PPG signals with Polar (ECG). (**a**) Recovering PPG with reference motion; (**b**) Synchronization between recovered PPG and golden standard ECG.

Figure 16a shows PPG signals together with the reduced motion and Figure 16b synchronization with the criterion standard method of Polar electrocardiogram (ECG).

### 3.2. Statistical Analysis of HR between OEPS and Commercial Devices

Statistical analysis of HR was performed using IBM SPSS Statistics 21 (SPSS Inc, Chicago, IL, USA). Statistical significance was accepted at *p* < 0.05. All participant characteristics are presented as mean ± standard deviation and standard error of the mean (*SEM*). Student t-tests were performed between the HR data from Polar and the OEPS to determine when a difference was evident between the two devices. The same test was implemented between Mio-alpha and the OEPS in order to validate the data extracted from PPG signals of the OEPS. The relationships between Polar, Mio-alpha and the HR outputs from the OEPS were examined via correlation (r) analysis (Pearson product moment). The agreement between the two devices was evaluated by means of using Bland Altman’s (BAA) 95% limits of agreement approach. Table 1 summarizes a comparison study between Polar and the OEPS.

**Table 1:** Bias, 95% limits of agreement (LOA) and correlation coefficient (r) of HR between Polar and the OEPS during Rest, Walking, Running and Cycling Activity, number of subjects (*n* = 16). Student paired t-tests for HR between Polar and the OEPS.

	Polar		OEPS		Bias	LOA ^−^	LOA ^+^	r	Intercept	Gradient
	Mean	SEM	Mean	SEM						
**Rest**	72	3	71	3	−1.13	−6	3	0.99	6.50	0.92
Treadmill (movement)	116	4	118	4	2.48	−14	19	0.96	2.98	0.95
4km/h ^a^	85	3	89	4	3.66	−18	26	0.70	36.42	0.55
7 km/h	117	3	118	3	1.56	−10	13	0.89	−4.55	1.03
8.5 km/h ^a^	144	4	148	4	2.33	−13	17	0.89	23.84	0.82
Treadmill (still)	119	5	118	5	−1.08	−23	20	0.93	6.74	0.95
4 km/h ^b^	88	3	89	4	1.15	−14	17	0.84	33.51	0.61
7 km/h ^c^	121	5	119	4	−2.83	−13	8	0.94	5.16	0.98
8.5 km/h ^c^	147	6	148	6	−1.75	−35	31	0.62	57.77	0.62
Cycling	135	3	133	3	−3.11	−21	15	0.93	5.64	0.98
1 kg	116	4	113	4	−2.81	−9	3	0.98	3.40	0.99
1.5 kg	129	5	126	5	−2.38	−12	7	0.97	10.33	0.94
2 kg ^a^	144	6	141	5	−2.53	−16	11	0.96	−15.86	1.13
2.5 kg ^a^	158	6	153	5	−4.80	−38	28	0.69	44.59	0.74

LOA^−^, Lower limits of agreement; LOA^+^, Upper limits of agreement. ^a^ n = 15; ^b^ *n* = 13; ^c^ n = 12.

**Figure 17:**
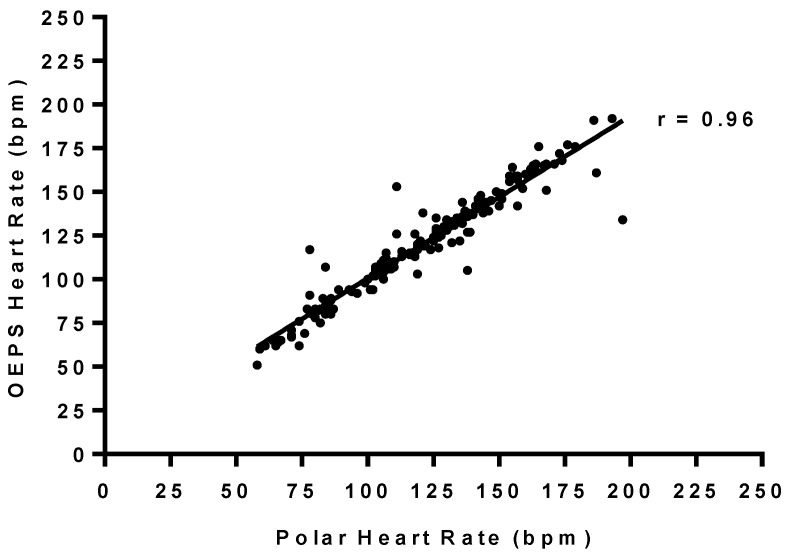
Correlation relationship between HR during rest and differing exercise modalities and intensities using Polar and the OEPS (r = 0.96, *p* < 0.0001).

**Figure 18:**
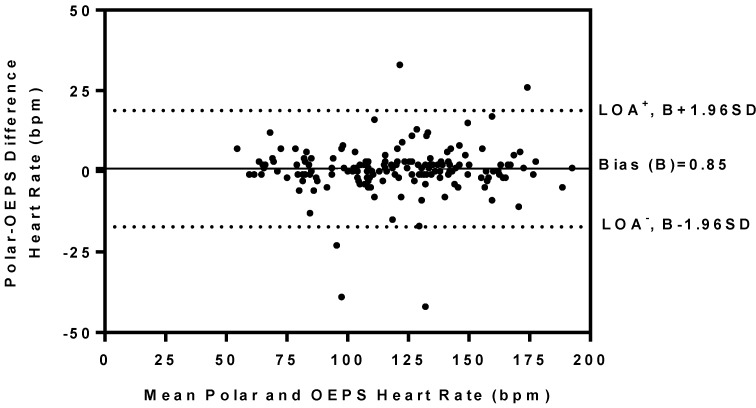
Bland-Altman plot shows differences in HR outputs recorded at rest and during differing exercise modalities and intensities using Polar and the OEPS. Mean bias (solid line) and limits of agreement (dashed line) are also shown.

Table 1 summarizes the mean values of HR presented by both Polar and the OEPS are comparable; a high linear association (r ≥ 0.96) was found between two measurements of HR as shown in Figure 17. No significant deference in HR was found between both devices (*p* = 0.73). The BAA plot of the difference (Polar-OEPS) against the mean value in HR is given by two methods ((Polar + OEPS)/2) is presented in Figure 18. The bias is B: 0.85 bpm, the standard deviation is SD: 9.20 bpm, and the limits of agreement (LOA) are in the range of −17.18 bpm to +18.88 bpm for lower and upper limits of agreement respectively. The 95% confidence intervals for the bias are B ± 1.96SD bpm for the lower (LOA^−^), and the upper (LOA^+^). All these intervals are reasonably narrow; as 95% of all (Polar-OEPS) data differences fall within the range of LOA.

**Figure 19:**
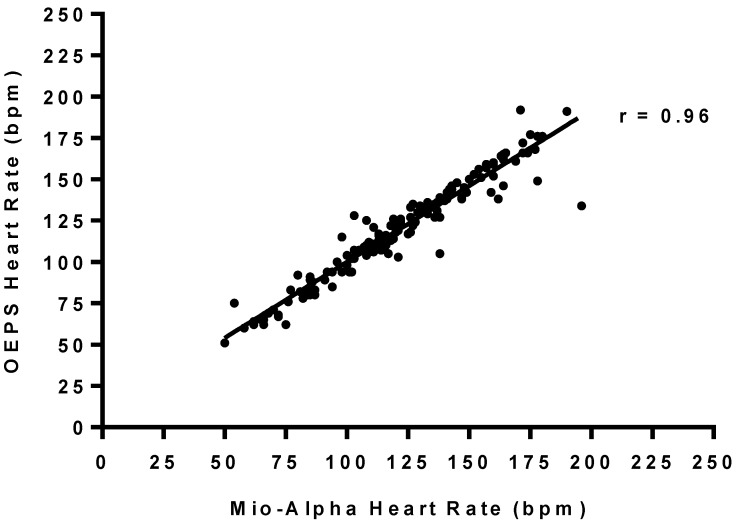
HR Correlation between Mio-Alpha and the OEPS (r = 0.96, *p* < 0.0001) during rest and differing exercise modalities and intensities.

**Figure 20:**
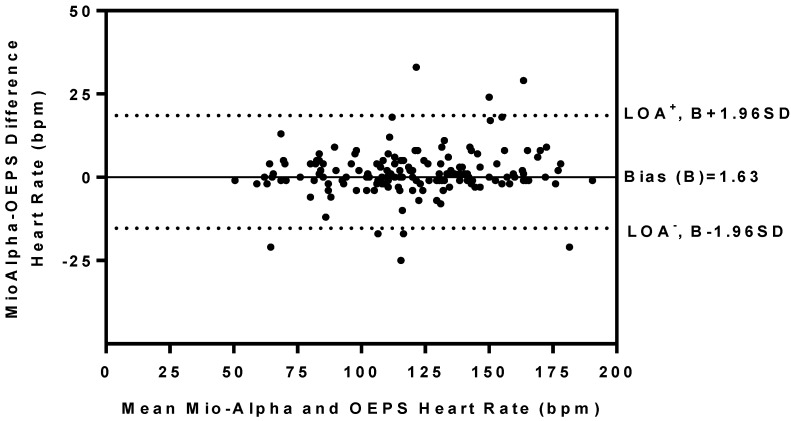
Bland-Altman plot shows differences in HR outputs recorded at rest and during differing exercise modalities and intensities using Mio-Alpha and the OEPS. Mean bias (solid line) and limits of agreement (dashed line).

**Figure 21:**
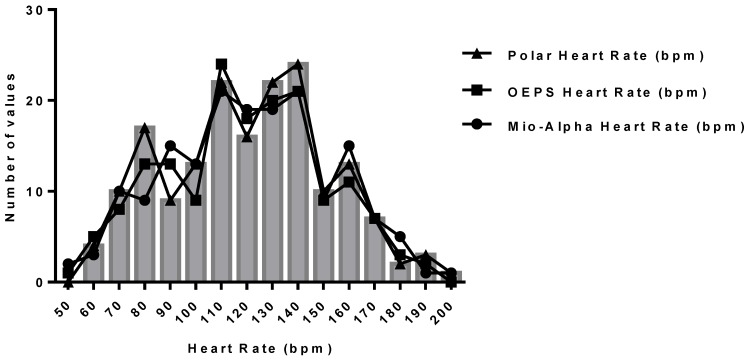
Histogram distribution for HR using Polar, Mio-Alpha and OEPS data.

The comparisons of HR between two measurement techniques, *i.e.*, Mio-Alpha and the OEPS were performed using the t-test for paired data as shown in Table 2. Pearson’s correlation analysis was also used to correlate quantitative variables (r ≥ 0.96), as an indicator of two techniques evolving in parallel in Figure 19. The test shows there is no significant difference as probability value (*p* = 0.67). The Bland-Altman method was used to compare the values of HR obtained by the OEPS technique and the commercial HR monitoring using Mio-Alpha watch. Figure 20 shows the bias B: 1.63 bpm, standard deviation SD = 8.62 bpm, lower and upper limits of agreement, −15.27 bpm and +18.58 bpm respectively. A histogram frequency distribution was used for all recorded data as shown in Figure 21.

**Table 2:** Bias, 95% limits of agreement (LOA) and correlation coefficient (r) of HR between Mio-Alpha and the OEPS during Rest, Walking, Running and Cycling Activity (16 subjects).

	Mio-Alpha		OEPS		Bias	LOA ^−^	LOA ^+^	r	Intercept	Gradient
	Mean	SEM	Mean	SEM						
Rest	71	3	71	3	0.18	−4	5	0.98	−8.63	0.90
Treadmill (movement)	119	4	118	4	2.48	−14	19	0.96	6.98	0.92
4 km/h	91	4	89	4	0.68	−8	9	0.96	−24.06	0.91
7 km/h	116	3	118	3	−2.37	−19	15	0.74	8.42	0.30
8.5 km/h ^a^	150	4	148	4	2.33	−13	17	0.89	−23.93	0.59
Treadmill (still)	120	5	118	5	1.39	−23	20	0.93	9.74	0.94
4 km/h ^b^	89	3	89	4	0.42	−13	14	0.89	−1.77	0.51
7 km/h ^d^	122	5	119	4	3.25	−10	17	0.92	−9.12	0.55
8.5 km/h ^d^	150	6	148	6	4.91	−21	31	0.76	−33.84	0.29
Cycling	135	3	133	3	2.47	−21	15	0.93	4.64	0.98
1 kg ^c^	113	3	113	4	1.00	−9	11	0.95	−7.20	0.68
1.5 kg ^a^	128	5	126	5	2.86	−7	13	0.96	−14.00	0.77
2 kg ^a^	142	5	141	5	−1.21	−5	8	0.98	−2.02	0.80
2.5 kg ^a^	157	6	153	5	−4.66	−29	39	0.63	−22.56	0.17

LOA^−^, Lower limits of agreement; LOA^+^, Upper limits of agreement. ^a^ *n* = 15; ^b^ *n* = 14; ^c^ *n* = 13; ^d^ *n* = 12.

## 4. Discussion

The algorithm for separating different wavelengths and detection of HR from PPG signals is efficient, despite the small SNR for both 650 nm and 870 nm wavelength illuminations during excessive movement. The APTRD for HR peak/trough detection works properly and this is evident because it calculates the HR accurately from the same waveform with different sample intervals. In addition, the APTRD provides valuable information such as the number of beats in a certain duration that could help in the determination of HRV to allow an assessment of cardiac nervous system by determining pulse to pulse interval (PPI) in a certain frequency domain. The results from the present study demonstrate the reliable PPG signals from the promising of the OEPS.

In this work, a six 532 nm LEDs was used to illuminate the designated tissue site due to its resistance against the corruption from a physical movement [42]. Moreover, the 532 nm wavelength LED with a narrower spectral band (±10 nm) could be useful to effectively illuminate the peripheral blood vessels and to capture the pulsatile dynamic variation (PDV).

The comparisons of HR between measurement methods of (Polar and the OEPS) and (Mio-Alpha and OEPS) were performed using the t-test. The results show there is no significant difference between methods (*p* > 0.05). The correlation analysis demonstrates a strong relationship in HR measured using the Polar and the OEPS as well as Mio-Alpha and the OEPS (both r = 0.96 and *p* < 0.0001).

The BAA method was applied to compare the values of HR obtained by the OEPS to the commercial devices, *i.e.*, Polar and Mio-Alpha. The bias in HR measured using the OEPS and both the Polar and Mio-Alpha was found to be low and the limits of agreement are acceptable. The histogram of differences between the measurements of two methods presents a normal distribution. There is a slight difference among the data due to an invalid data set presented on the sensors (Polar, Mio-Alpha and the OEPS). The main reason for the distortion of the signal captured by the OEPS was inadequate attachment of the sensor; thus resulting in a misalignment of the area of interest.

## 5. Conclusions

This work introduced an OEPS together with an opto-electronic system and an algorithm, *i.e.*, APTRD/MAR for accurate detection of HR and eliminating noise signals caused by motion artefacts respectively. Both the OEPS and the APTRD/MAR indeed enhance the quality of PPG signal to determine HR during route activities or an exercise. The APTRD converts the PPG signals collected during a cycling and treadmill running exercise into a HR value. The commercial devices (Polar and Mio-Alpha) were used to validate the OEPS derived HR. The good agreement analysis shows the comparability of the both techniques of the OEPS and commercial methods in the evaluation of HR. The limitation of invalid data or signal error that appears due to misalignment could be overcome by the means of a proper packaging for the OEPS. The outcome from the proposed OEPS indicates a new paradigm of opto-physiological monitoring technique as ideally suited to be incorporated into wearable devices.
